# Toxins and Other Bioactive Metabolites in Deep Chlorophyll Layers Containing the Cyanobacteria *Planktothrix* cf. *isothrix* in Two Georgian Bay Embayments, Lake Huron

**DOI:** 10.3390/toxins13070445

**Published:** 2021-06-27

**Authors:** Arthur Zastepa, Todd R. Miller, L. Cynthia Watson, Hedy Kling, Susan B. Watson

**Affiliations:** 1 Environment and Climate Change Canada, Canada Centre for Inland Waters, 867 Lakeshore Road, Burlington, ON L7S 1A1, Canada; linet.watson@canada.ca; 2 Joseph J. Zilber School of Public Health, University of Wisconsin-Milwaukee, Milwaukee, WI 53211, USA; millertr@uwm.edu; 3 Algal Taxonomy and Ecology Inc., P.O. Box 761, Stony Mountain, MB R0C 3A0, Canada; hedy.kling8@gmail.com; 4 School of Graduate Studies, Environmental and Life Sciences, Trent University, Peterborough, ON K9L 0G2, Canada; sboydwatson@gmail.com

**Keywords:** deep-chlorophyll layers (DCLs), cyanobacterial toxins, *Planktothrix*, allelopathy, bioactive metabolites, hypoxia, Georgian Bay

## Abstract

The understanding of deep chlorophyll layers (DCLs) in the Great Lakes—largely reported as a mix of picoplankton and mixotrophic nanoflagellates—is predominantly based on studies of deep (>30 m), offshore locations. Here, we document and characterize nearshore DCLs from two meso-oligotrophic embayments, Twelve Mile Bay (TMB) and South Bay (SB), along eastern Georgian Bay, Lake Huron (Ontario, Canada) in 2014, 2015, and 2018. Both embayments showed the annual formation of DCLs, present as dense, thin, metalimnetic plates dominated by the large, potentially toxic, and bloom-forming cyanobacteria *Planktothrix* cf. *isothrix*. The contribution of *P.* cf. *isothrix* to the deep-living total biomass (TB) increased as thermal stratification progressed over the ice-free season, reaching 40% in TMB (0.6 mg/L at 9.5 m) and 65% in South Bay (3.5 mg/L at 7.5 m) in 2015. The euphotic zone in each embayment extended down past the mixed layer, into the nutrient-enriched hypoxic hypolimnia, consistent with other studies of similar systems with DCLs. The co-occurrence of the metal-oxidizing bacteria *Leptothrix* spp. and bactivorous flagellates within the metalimnetic DCLs suggests that the microbial loop plays an important role in recycling nutrients within these layers, particularly phosphate (PO_4_) and iron (Fe). Samples taken through the water column in both embayments showed measurable concentrations of the cyanobacterial toxins microcystins (max. 0.4 µg/L) and the other bioactive metabolites anabaenopeptins (max. ~7 µg/L) and cyanopeptolins (max. 1 ng/L), along with the corresponding genes (max. in 2018). These oligopeptides are known to act as metabolic inhibitors (e.g., in chemical defence against grazers, parasites) and allow a competitive advantage. In TMB, the 2018 peaks in these oligopeptides and genes coincided with the *P.* cf. *isothrix* DCLs, suggesting this species as the main source. Our data indicate that intersecting physicochemical gradients of light and nutrient-enriched hypoxic hypolimnia are key factors in supporting DCLs in TMB and SB. Microbial activity and allelopathy may also influence DCL community structure and function, and require further investigation, particularly related to the dominance of potentially toxigenic species such as *P.* cf. *isothrix*.

## 1. Introduction

Deep chlorophyll layers (DCLs) are ecologically important but often overlooked phenomena in many deep lentic ecosystems. DCLs can account for a significant fraction of pelagic primary productivity, play a key role in biogeochemical cycling of nutrients, and influence the vertical movement of micrograzers [1,2,3,4,5,6,7]. DCLs are likely far more prevalent than reported, as most sampling efforts concentrate on surface water layers. Investigations of large and deep Canadian waters such as the Great Lakes, where offshore DCLs have been observed as deep as ca. 50 m, have documented assemblages dominated by diatoms, picoplankton, and cryptophytes, along with the presence of other flagellated autotrophs/mixotrophs (e.g., chrysophytes, dinoflagellates) [8,9,10,11]. Previous studies have identified the importance of light (euphotic depth, Z_eu_) and thermal stratification in predicting the depth and thickness of deep-living phytoplankton communities, but less is known about the influence of chemical (e.g., nutrient) gradients and even less about the chemical ecology of this deep-living biota [4,7,12,13,14]. Several mechanisms can produce chemical gradients conducive to DCL formation and progression, including upwelling, inter-flow, groundwater, and diffusion from surficial sediments, i.e., internal loading (e.g., [15]). Internal loading often occurs in productive, stratified systems within a hypoxic hypolimnion and can provide a supply of bioavailable dissolved nutrients that are directly accessible to deep-living phytoplankton particularly in shallow waterbodies where Z_mix_ < Z_eu_ [16]. Studies of DCLs in smaller and shallower (<30 m) lakes in Canada report flagellated autotrophs/mixotrophs, e.g., chrysophytes as being the most prominent [1,2,6,17]. However, a more recent study documents DCLs dominated by large cyanobacteria in two nearshore embayments of eastern Georgian Bay (Lake Huron, Ontario, Canada) (e.g., [16]).

Earlier work along the eastern coast of Georgian Bay reported DCLs in a number of sheltered embayments of intermediate size and depth (<30 m), many of which regularly undergo thermal stratification and establish pronounced chemical gradients with depth, [16]. Building on this research, we carried out two nearshore surveys (2014 and 2015) of 15 embayments along this coastline to further investigate the presence of DCLs, and selected two of these waterbodies for more focused work. In 2015, detailed measurements were done in these two embayments to characterize the seasonality and vertical structure of each documented DCL and its phytoplankton composition as well as the coincident physicochemical and nutrient gradients relevant to phytoplankton physiology. We also measured a suite of bioactive metabolites that can play a role in cyanobacterial dominance (the cyanobacterial toxins microcystins, nodularin, anatoxins, saxitoxins, and cylindrospermopsins, and several other metabolic inhibitors known to act at the food web level, anabaenopeptins, cyanopeptolins, and microginins) [13]. Samples from the DCL sites were also obtained in 2018 for additional investigations of these bioactive metabolites, including genetic screening. While the ‘toxins’ have been widely measured, studies of other metabolic inhibitors have been largely limited to Europe with a few recent reports from North America [13,18,19]. To our knowledge, however, none of these other bioactive metabolites have been characterized in DCLs.

## 2. Results and Discussion

**Figure 1:**
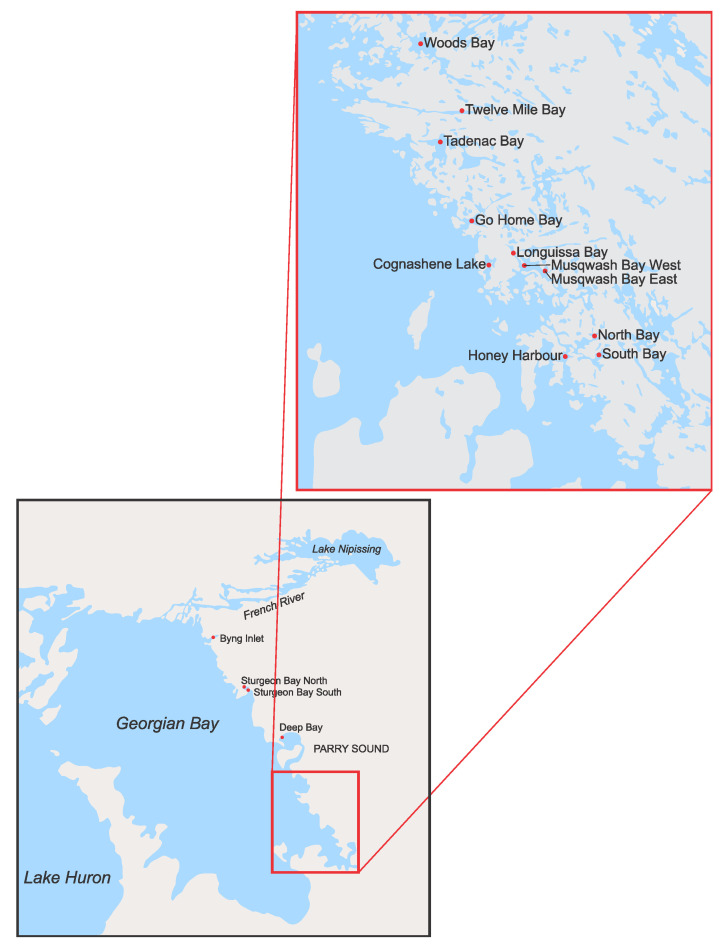
Locations of study sites in Georgian Bay, Lake Huron (Ontario, Canada) denoted by red circles.

**Figure 2:**
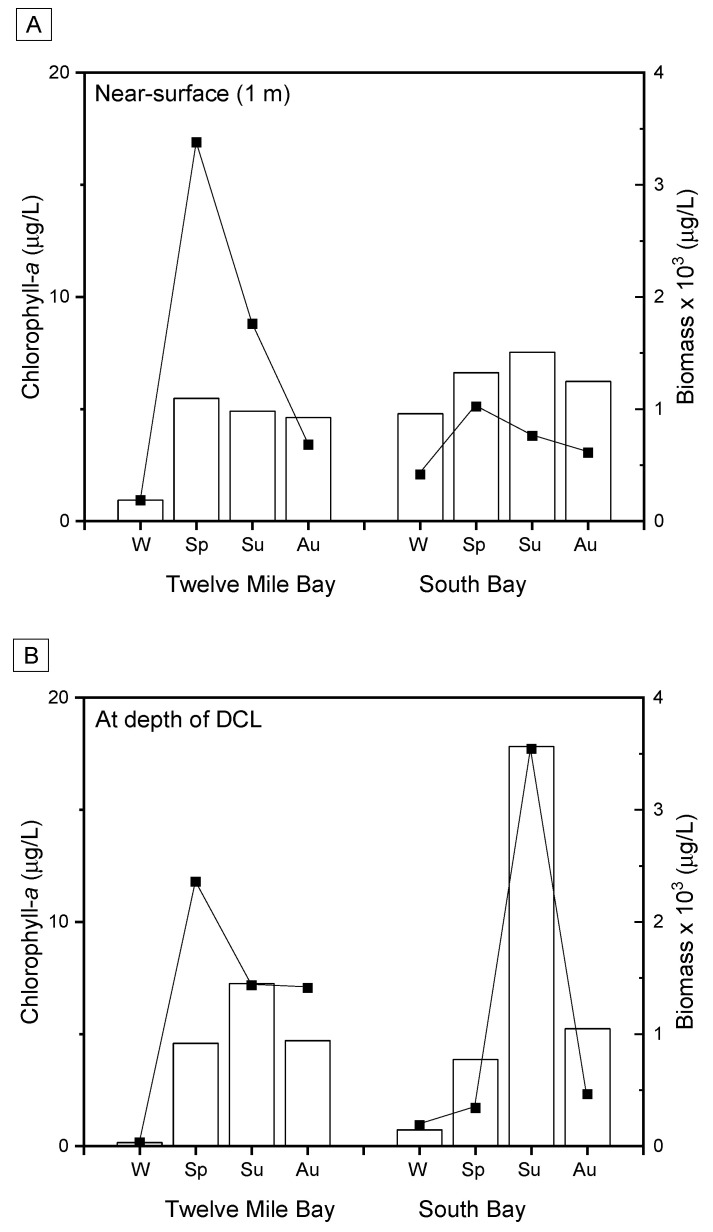
Seasonal (W, winter; Sp, spring; Su, summer; Au, autumn) changes in total phytoplankton biomass (line series) and extracted chlorophyll-*a* concentration (bars) in 2015. Near-surface (1 m) water column measurements are in panel (**A**) and at the depth of the recurring metalimnetic peaks, i.e., deep chlorophyll layers (DCLs) (~9 m in Twelve Mile Bay (TMB) and ~7 m in South Bay (SB), see Table 1 and Figure 3) are in panel (**B**). In the absence of DCLs during winter (W) and spring (Sp), i.e., during isothermal conditions, the sample was taken at the depth corresponding to summer DCL peak for comparison, i.e., ~9 m in TMB and ~7 m in SB. See Materials and Methods for sampling dates.

**Figure 3:**
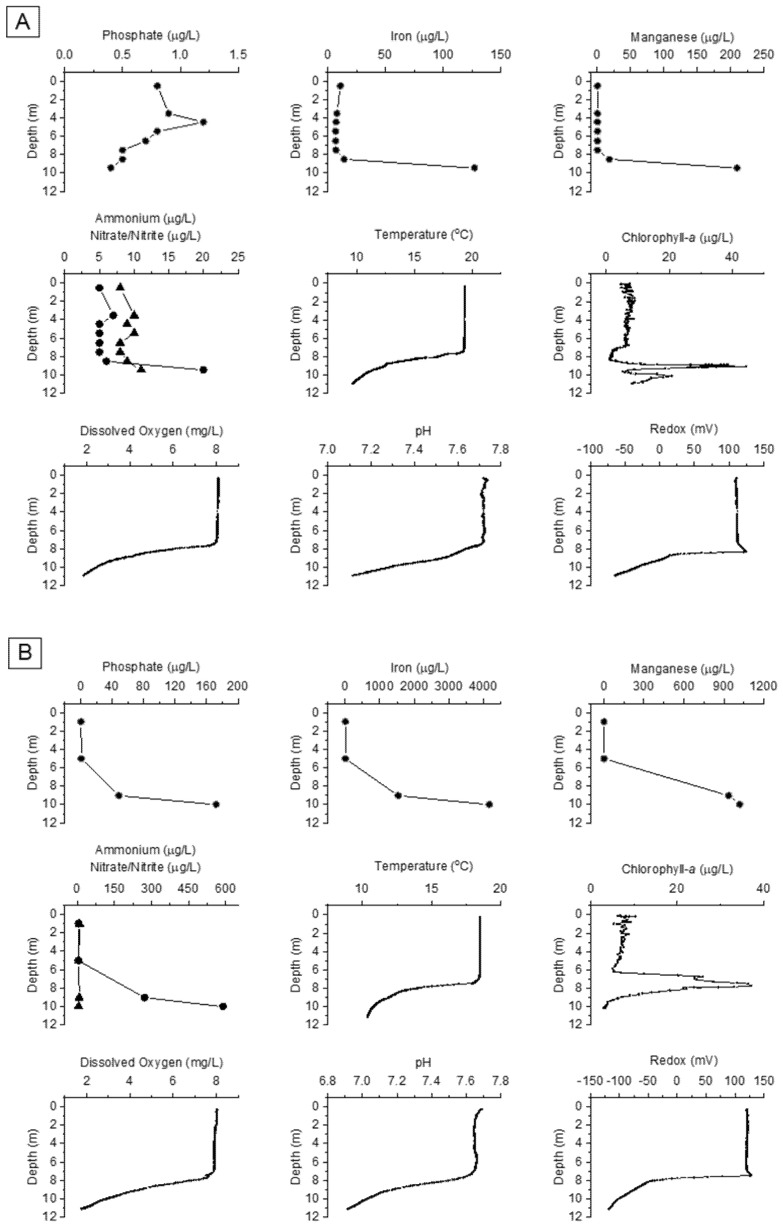
Water column profiles in Twelve Mile Bay (panel **A**) and South Bay (panel **B**) for: phosphate (PO_4_), iron (Fe), manganese, ammonium (NH_4_) (●), nitrite/nitrate (NO_2/3_) (▲), temperature, chlorophyll-*a*, dissolved oxygen, pH, and redox in summer (August) of 2015. See Materials and Methods section for measurement and analytical protocols used.

**Figure 4:**
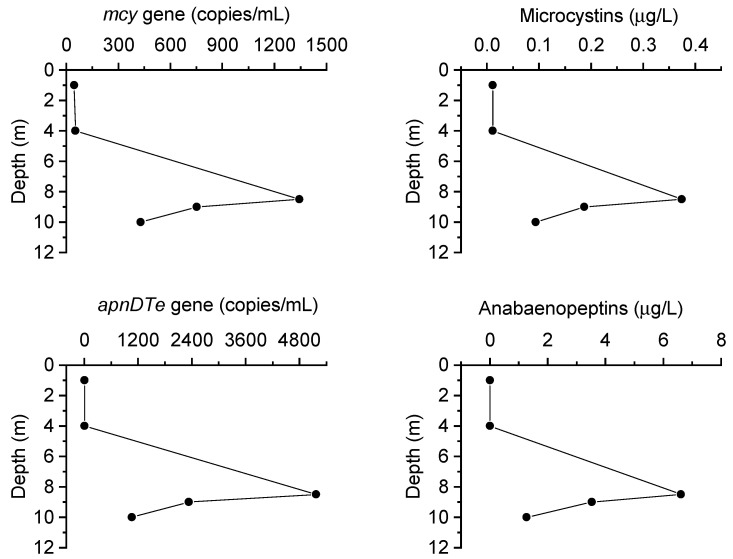
Vertical water column gradients of bioactive metabolites (microcystins and anabaenopeptins) produced by cyanobacteria and corresponding genes (*mcy* and *apnDTe*, respectively) in Twelve Mile Bay in summer (August) of 2018. Note that higher concentrations of these bioactive metabolites (and their genes) co-occur with biomass peaks, notably of *Planktothrix isothrix*, at depth (Table S1, 2018 data).

DCLs were documented in 2 of 15 embayments, Twelve Mile Bay (TMB) and South Bay (SB), during a spatial survey conducted in 2014 (Figure 1, Table 1). In 2015, the full survey was repeated in addition to a more detailed seasonal sampling of TMB and SB (Figure 2) where profiles and discrete depth samples were collected to characterize the vertical structure of phytoplankton composition (Table S1 in Supplementary Materials), the coincident physicochemical and nutrient gradients (Figure 3, Table 2), and a suite of bioactive metabolites (Table 3). In 2018, additional samples were collected from the two DCL sites to confirm previous results on bioactive metabolites (Table 3) and construct vertical water column profiles of corresponding genes (Figure 4).

### 2.1. Spatiotemporal Changes in Phytoplankton Composition in Near-Surface and Deep Chlorophyll Layers in Twelve Mile Bay

Overall, TMB phytoplankton showed seasonality and a near-surface biomass, which was composed of a diverse community of flagellates, diatoms and picoplankton, typical of many meso-oligotrophic zones of the Great Lakes (e.g., [20]). While some of these near-surface taxa were also present in the deeper strata, DCLs were often composed of a distinct community, and dominated by trichomes of the cyanobacteria *P.* cf. *isothrix,* which was never observed in near-surface samples. In the summer of 2014, TMB was stratified with a distinct DCL at 10 m depth at the metalimnion, exceeding a total biomass (TB) of 1600 µg/L, almost double that in the epilimnion (Table S1). The DCL was dominated by the buoyancy-regulating cyanobacteria *Planktothrix* cf. *isothrix* (>1300 µg/L, 80% TB), with pennate diatoms (7% TB) and cryptophytes (4% TB) also abundant (Table S1). This deep-living community differed markedly from the epilimnetic assemblage, which was dominated by large flagellates—dinoflagellates (13% TB), cryptophytes (24% TB) and colonial chrysophytes (10% TB).

By autumn 2014, lake turnover brought on isothermal conditions and a more uniform vertical distribution of TB (~1000 µg/L) and composition. Both near-surface (1 m) and deep (10 m) samples had a diverse community dominated by large pennate diatoms (*Fragilaria crotonensis*, up to ~40% TB) and chrysophytes (up to ~40% TB; *Dinobryon sertularia*, *Synura* sp., ochromonads). The deep-living population of *P.* cf. *isothrix* seen during the summer was reduced significantly (<50 µg/L, 4% TB) and, again, this species was not observed near-surface. We also noted a small population of the nuisance flagellate *Gonyostomum semens* (<40 µg/L, 4% TB) near-surface. While commonplace in low numbers in softwater lakes, this raphidophyte can produce periodic blooms in boreal lakes (e.g., [21]) and outbreaks have been linked to high Fe availability [22].

During the winter ice cover of 2015, low near-surface biomass (<200 µg/L) was dominated by chrysoflagellates (55% TB), cryptophytes (24% TB) and dinoflagellates (3% TB), with low numbers of cyanobacteria also present (7% TB; *Microcystis* sp., *Planktothrix* sp.) (Figure 2, Table S1). Near-bottom (10 m) samples showed a sparse biomass (<40 µg/L), composed of mixotrophic chrysophytes (16% TB) and the large heterotrophic dinoflagellate *Gymnodinium helveticum* (14% TB), along with filaments of *Planktothrix* cf. *isothrix* (10% TB) and the diatom *Aulacoseira subarctica* (7% TB).

In spring 2015, there was a significant increase in biomass, particularly near-surface (>3300 µg/L TB) (Figure 2, Table S1). Near-surface (1 m) and deep (10 and 11.5 m) samples were all dominated by the large diatom *Fragilaria crotonensis* (up to 80% TB); large chrysoflagellates (up to 10% TB) were also important while cryptophytes (10% TB) were only present closest to the bottom (11.5 m). Low numbers of the colonial cyanobacteria *Aphanothece minutissima* and *Microcystis* sp. were present in the two deeper samples, but *Planktothrix* was not detected.

**Figure A1:**
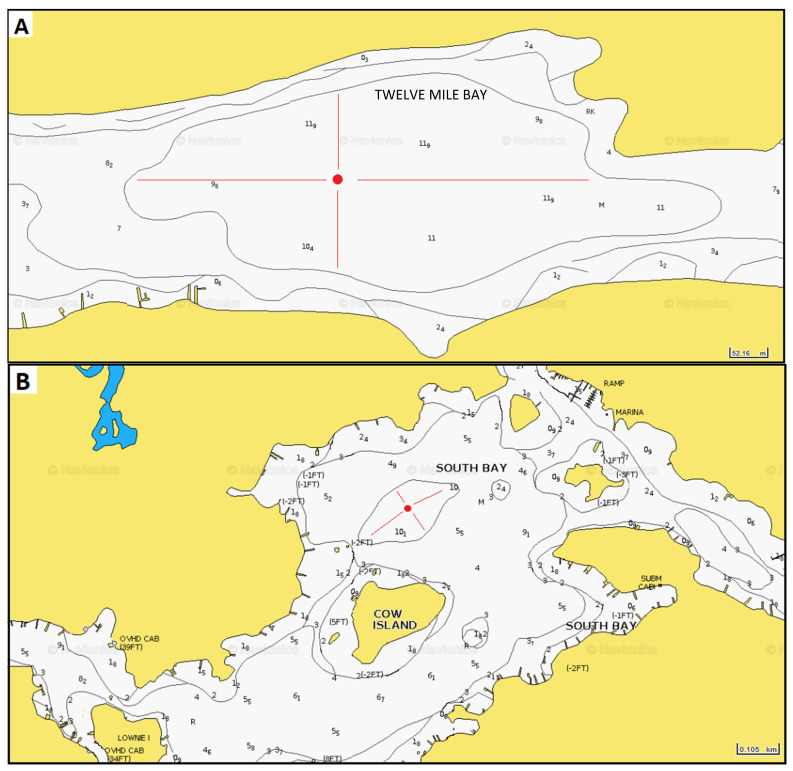
Location within (**A**) Twelve Mile Bay (TMB), scale 52.16 m and (**B**) South Bay (SB), scale 0.105 km, in Georgian Bay, Lake Huron, Ontario, Canada where each of the deep-chlorophyll layers were sampled, denoted by a filled, red circle. Three-dimensional mapping by Fluoroprobe© revealed that each DCL extended horizontally to the end of the first contour demarcated on the map (~9 m in TMB and ~10 m in SB, approximate extent indicated by red lines) and therefore each of these contours were used to estimate the elliptical area over which each of the DCLs existed. Maps reproduced from Navionics Web API v2, www.navionics.com accessed on January 2021.

Thermal profiles in summer of 2015 revealed a stratified water column, with a mixed/epilimnetic layer (down to ~7 m) overlaying a steep thermocline to the bottom and no discernible hypolimnion (Figure 3). Epilimnetic biomass (>1700 µg/L) was again dominated by diatoms (65% TB; notably *F. crotonensis*), with large thecate dinoflagellates (13% TB) also being important (Table S1). Cyanobacteria constituted only a small fraction (14%) of this biomass, largely as colonial picocyanobacteria (*Aphanocapsa* sp., *Aphanothece minutissima*) and a few trichomes of the diazotrophic cyanobacteria *Dolichospermum* cf. *fuscum*. At depth, the phytoplankton community segregated into two distinct DCLs (~1 m apart), each showing strong peaks (>20 µg/L) in chlorophyll-*a* fluorescence (Figure 3). The more prominent DCL was concentrated within a ~1 m thick layer at the meta-hypolimnetic boundary (~9 m deep). FP mapping estimated that this DCL extended horizontally over an elliptical area of almost 0.2 km^2^ until the end of the first (~9 m) contour (Figure 3, Figure A1). This biomass peak (>1400 µg/L) was dominated by cryptophytes (50%TB; *C. reflexa*), diatoms (13% TB; possibly settling from the epilimnion) and *P.* cf. *isothrix* (8% TB) (Figure 2, Table S1). While water samples from the smaller DCL at 10 m could not be obtained, FP fluorescence indicated an assemblage dominated by “brown/golden” phytoplankton (e.g., dinoflagellates, diatoms, chrysophytes) (Figure 3).

In the autumn of 2015, the water column showed a deepened mixed/epilimnetic layer and a single metalimnetic DCL at ~9 m. Near-surface biomass had decreased to <700 µg/L and was dominated by large mixotrophic and non-mixotrophic chrysophytes (38% TB) and thecate dinoflagellates (14% TB) (Figure 2, Table S1). Small populations of colonial picocyanobacteria were also present but *Planktothrix* was not observed. The DCL contained double the near-surface biomass (>1400 µg/L) and was dominated by *Dinobryon* sp. (70% TB); *Peridinium* sp. (9% TB) and *P.* cf. *isothrix* (8% TB) were also important. A sample taken just above the sediments at 10 m showed a significantly lower total biomass (<700 µg/L) but an increased proportion of *P.* cf. *isothrix* (40% TB) (Figure 2, Table S1).

Although *P.* cf. *isothrix* was absent near-surface in TMB throughout all sampling, it was present in samples from both the sediment-water interface and surficial sediments throughout the seasons. Sediments—particularly those collected in winter—yielded (on qualitative inspection) viable trichomes of *P.* cf. *isothrix* under laboratory conditions simulating the summer metalimnion (Z8 media, <15 °C and <50 µmol m^−2^ s^−1^). This suggests overwintering filaments could act as a reservoir of viable cells for the metalimnetic populations of this species. Recruitment of viable, vegetative cyanobacteria cells overwintering in sediments has been observed elsewhere with bloom-forming cyanobacteria, notably *Oscillatoria* (syn. *Planktothrix*), *Dolichospermum* (syn. *Anabaena*) and *Microcystis* [23,24,25,26]; however, in these cases, the benthic cyanobacteria were implicated in supporting surface blooms rather than DCLs. In TMB, our observations suggest that the sediments serve as a reservoir of viable *P.* cf. *isothrix* cells which seed the DCLs, and to our knowledge remain at depth and do not appear in any detectable numbers at or near the surface. While our evidence is supportive, a more direct measurement of this recruitment process is needed to confirm our hypothesis that sediment seeding may play an important role in the origins, seasonal dynamics, and fate of cyanobacteria-dominated DCLs in TMB.

### 2.2. Spatiotemporal Changes in Phytoplankton Composition Near-Surface and within Deep-Chlorophyll Layers in South Bay

In summer of 2014, SB was stratified with a distinct metalimnetic DCL at 6.5 m, reaching >60 µg/L chlorophyll-*a*—six times that measured near-surface (1 m)—of which ~87% was derived from cyanobacteria (based on FP fluorescence). Although a detailed taxonomic identification and enumeration of the summer 2014 DCL was not possible, results from subsequent years (reported below) suggest *P.* cf. *isothrix* as a prominent and recurring member of these deep phytoplankton communities in SB. Near-surface (1 m) summer biomass in 2014 was ~1000 µg/L, and dominated by chrysophytes (35% TB), dinoflagellates (10% TB), and picocyanobacteria (7% TB) (Table S1). Large cyanobacteria were present but in very low biomass (~3% TB; notably *Dolichospermum*).

By autumn 2014, lake turnover brought on isothermal conditions and biomass had concentrated near-surface, increasing over three times to ~3000 µg/L; dominated by large diazotrophic cyanobacteria (*Aphanizomenon flos aquae* complex, 45% TB; *Dolichospermum planctonicum*, 4% TB), with *Euglena* sp. (9% TB), *Aulacoseira ambigua* (9% TB), and *Plagioselmis nanoplanktica* (8% TB) also being abundant (Table S1).

During the winter ice cover of 2015, biomass near-surface (>400 µg/L at 1 m) was dominated by large colonial, scaled chrysoflagellates (*Synura* sp., 77% TB), with small populations (<5% each) of dinoflagellates and picocyanobacteria (Table S1). In contrast, a much lower biomass at ~8 m depth (<200 µg/L) was composed of picocyanobacteria (*Synechococcus spp*., 45% TB), chrysophytes (37% TB), and dinoflagellates (9% TB).

By spring of 2015, under isothermal conditions, near-surface (1 m) biomass exceeded 1000 µg/L; dominated by a variety of chrysoflagellates (37% TB), cryptophytes (17% TB; mostly *C. reflexa*) and the large, horned dinoflagellate *Ceratium furcoides* (15% TB) (Figure 2, Table S1). At 5.5 m depth, a comparable biomass (>1000 µg/L) had an assemblage of scaled chrysophytes (36% TB; e.g., *Chrysosphaerella longispina*) and large dinoflagellates (*Ceratium* sp., 21% TB) with a few diazotrophic cyanobacteria (*Dolichospermum lemmermanni*, *Aphanizomenon flos-aquae*) also present. Near-bottom (10.5 m) samples had a less biomass (<400 µg/L), composed mostly of *Cryptomonas* spp. (32% TB), *Chlamydomonas* sp. (14% TB), and *P.* cf. *isothrix* (9% TB).

Profiles in summer of 2015 revealed a stratified water column with near-surface (1 m) biomass (~800 µg/L) dominated by filamentous diatoms (34% TB; *Aulacoseira*), thecate dinoflagellates (15% TB)*,* and the diazotroph *D. planktonicum* (12% TB) (Figure 2, Table S1). A distinct and sizable DCL (>3500 µg/L) was concentrated within a ~1 m thick layer at the meta-hypolimnetic boundary (~7 m). FP mapping estimated that the DCL extended horizontally over an elliptical area of almost 0.03 km^2^, extending over an area bounded by the ~10 m depth contour (Figure 3, Figure A1). This deep biomass peak was dominated by *P.* cf. *isothrix* (65%); other cyanobacteria were also present (*Aph. flos-aquae* complex, 6% TB; *Microcystis novacekii*, 3% TB) along with dinoflagellates (7% TB) and diatoms (4% TB).

By autumn 2015, lake turnover brought on isothermal conditions and a more uniform vertical distribution of phytoplankton, i.e., no DCL was observed. A similar sized surface biomass (~600 µg/L) showed a shift in community composition from the summer assemblage towards an increased proportion of diazotrophic cyanobacteria (*D. planktonicum*, 32% TB; *Aph. flos-aquae*, 6% TB) and a decrease in the filamentous diatom *Aulacoseira* (16% TB) (Figure 2, Table S1). In the near-bottom sample (~9 m), a comparable biomass (~500 µg/L) showed similar composition (*Aulacoseira*, 17% TB; *D. planktonicum*, 15% TB). As seen in summer, *P.* cf. *isothrix* was again present at depth but at comparably lower proportion (9%), but other cyanobacteria (*Pseudanabaena* sp. 8% TB; *Aph. flos*-*aquae* complex, 7% TB) were also observed.

We note that while numerous other studies have reported DCLs that have been mixed into the epilimnion by physical processes such as turnover, storms, or artificial aeration/mixing and subsequently manifested as ephemeral surface blooms [4,18,27,28,29,30,31,32], there have been no reports of surface blooms in TMB nor SB near the locations of the DCLs, despite the high density of cottagers in the area. This suggests that the thick metalimnetic plates of *Planktothrix* remain essentially segregated from the surface community and are seeded and sustained by the physicochemical conditions in the bottom layers and sediments.

### 2.3. Light and Pigmentation

**Figure A2:**
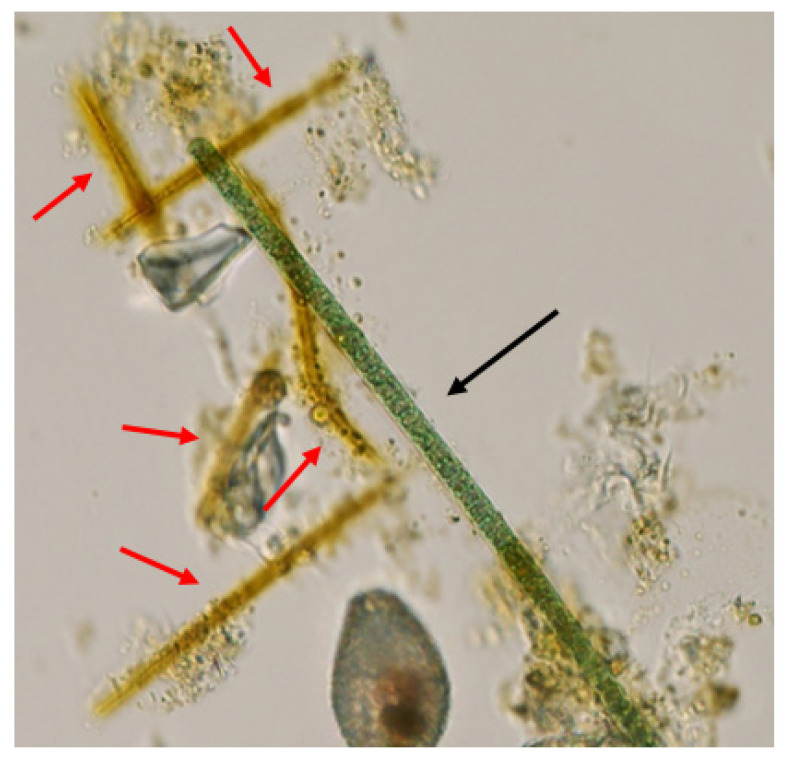
Microscopic imaging of water sample at the DCL (~9 m) in Twelve Mile Bay showing *Planktothrix* cf. *isothrix* (black arrow) and *Leptothrix* with Fe oxide precipitates (five red arrows). Images are not to scale and are only for representative purpose.

Light is a major factor which often controls phytoplankton vertical structure and the formation of deep-living communities (e.g., [7]), and many taxa modify or supplement their effective light regimes via vertical migration, mixotrophy, and photoadaptation. The abundance of large flagellated mixotrophs in the deep layers in TMB and SB (Table S1) suggests that motility and bactivory may provide access to alternative supplies of energy and nutrients (e.g., [17]); however, these photophagotrophs rarely predominated the DCLs in these embayments. PAR measures showed that the DCLs were positioned within the euphotic zone (Table 1), and we therefore conclude that in situ light levels were sufficient to support the development of *P.* cf. *isothrix*, i.e., light availability was not a primary factor limiting these DCL communities (e.g., [10,33]). Although current literature suggests phycoerythrin-rich *P. rubescens* is more often the predominant *Planktothrix* species in deep-living maxima, the predominant species *P.* cf. *isothrix* remained green-pigmented, suggesting minimal cellular phycoerythrin (Figure 2, Figure A2). We are aware of only one other published report of a DCL dominated by green-pigmented *Planktothrix* [3] although broader geographical surveys are needed [9,14,34,35,36,37,38].

### 2.4. Nutrients and Physicochemical Conditions

In SB, DCLs dominated by *P.* cf. *isothrix* were located at the metalimnion where sufficient PAR intersected with elevated levels of major nutrients and trace metals, particularly PO_4_, dissolved inorganic nitrogen (DIN) and dissolved Fe (Table 1, Table 2, Figure 3). Vertical gradients of these chemical constituents increased with depth through the hypolimnion, indicative of internal loading from surficial sediments [16,39,40,41]. PO_4_, Fe, NH_4_, Mn and Co concentrations were up to three orders of magnitude higher near the sediments as conditions became hypoxic (dissolved oxygen <2 mg/L). Compared to TMB, bottom concentrations were generally higher and gradients were more pronounced in SB—which also exhibited a lower redox potential in the hypolimnion, particularly near sediments (−120 mV vs. −65 mV in TMB). Although similar gradients with depth through the hypolimnion were also evident in TMB for most measures taken, they were not as pronounced as in SB and this, along with markedly lower PO_4_, is consistent with the significant difference in DCL biomass between the two embayments (Figure 2 and Figure 3). Interestingly, the sheathed bacteria *Leptothrix* were observed in the DCL of TMB but not SB (Figure A2). These chemoorganotrophs exploit DOC-rich environments in the presence of Fe^2+^ and Mn^2+^, which they precipitate as ferrous hydroxide and manganese oxide at the interface between oxic/anoxic zones, often co-precipitating PO_4_ and making it inaccessible to phytoplankton (e.g., [42,43]). Their PO_4_-precipitating activity in TMB may, to some extent, account for the minimal change in PO_4_ with depth despite conditions conducive to internal loading but further study to quantify the significance of this mechanism is needed. Differences in sediment chemistry (e.g., sulphate/sulphide or aluminum) could also account for the contrasting PO_4_ profiles but this has not yet been characterized nor quantified in the two embayments [39,40,41].

There is a considerable body of evidence to indicate that the development of opposing and intersecting vertical gradients of light and nutrients is strongly correlated with DCL formation in stratified, meso-oligotrophic systems [4,5,7,15,34,44]. Our survey showed that stratified Georgian Bay embayments lacking these opposing and intersecting vertical gradients also lacked discernible DCLs. Some, such as Sturgeon Bay, had a hypoxic hypolimnion and associated gradient in nutrients that reached the metalimnion but did not have sufficient light penetration to support a DCL, i.e., Z_eu_ << Z_mix_ (Table 1). Others, such as Cognashene Lake (actually an embayment), had sufficient light at the metalimnion (Table 1) and elevated nutrients in the hypoxic hypolimnion (e.g., >1400 µg/L Fe, >600 µg/L Mn, >200 µg/L NH_4_) but it was unclear if these opposing gradients intersected as we lacked sufficient resolution with depth. We speculate that the lake’s greater depth and thicker hypolimnion rendered the hypolimnetic nutrients inaccessible to phytoplankton at sufficient PAR (Table 1); however, with more stable and prolonged periods of stratification, and associated hypoxia, as expected with climate change, the nutrient gradient would presumably strengthen.

Consistent with our observations, others have observed metalimnetic DCLs most commonly occuring at depths of less than 15 m, particularly those dominated by bloom-forming cyanobacteria [4,5,7,31,34,38,44,45,46]. Although significant DCLs dominated by potentially toxic cyanobacteria such as *Planktothrix* have been reported at depths below 15 m (e.g., [47]), this appears relatively rare [7,46]. DCLs existing deeper than 15 m (close to 30 m depth) are generally dominated by picoplankton, flagellated autotrophs/mixotrophs (e.g., large colonial chrysophytes, dinoflagellates), and diatoms [9,10,11,15]. The occurrence of these deeper DCLs has been documented in much larger (>19,000 km^2^) and deeper systems (>60 m) including offshore Lake Ontario [10,11], Lake Michigan, Lake Superior, and Lake Huron [8,9] and in marine systems (e.g., [15]). The significant depth of these systems and corresponding thickness of the hypolimnion suggests upwelling of bottom waters [15] and recycling from biomass [1,6,48] as more likely sources of nutrients contributing to the DCLs—this, in contrast to the direct access metalimnetic DCLs in the relatively shallower TMB and SB have to the nutrient gradient generated from internal loading at the sediment-water interface. Although opposing and intersecting physicochemical gradients in nutrients and light are considered key drivers of DCL biomass formation in general; their effects on phytoplankton community composition and activity need further investigation.

### 2.5. Toxins Produced by Cyanobacteria

Over the course of sampling (2014, 2015, and 2018), near-surface summer samples from 5 of 15 Georgian Bay embayments showed both detectable levels of microcystins and the genetic potential to produce these hepatotoxins (positive for *mcy* gene). The highest concentration observed in any sample (0.4 µg L^−1^ in 2018) was well below the Canadian guidelines for safe drinking and recreational water (<1.5 µg/L; <20 µg/L respectively; Health Canada 2017) (Table 3, Figure 4). MC-LA was the most frequently observed microcystin variant and also present at higher concentrations than MC-LR and MC-RR. This is consistent with other reports suggesting MC-LA is more prevalent in systems with lower trophic status (e.g., [13,49]). In both TMB and SB, both the microcystin gene (*mcy*) and the microcystin toxins were present at the metalimnetic DCLs and in the hypolimnion. Based on our 2018 measurements, the *Planktothrix*-dominated DCL in TMB coincided with peaks in microcystin concentration and corresponding gene copies (*mcy*) (Figure 4, Table S1) suggesting these cyanobacteria as the primary source. Other species of *Planktothrix* are known for the production of a wide range of bioactive metabolites including microcystins [18]; however, production by *P.* cf. *isothrix* has yet to be directly confirmed.

The presence of the *mcy* gene and trace amounts of microcystin in the hypolimnion of Tadenac Bay is also noteworthy (Table 3) as this embayment is relatively unimpacted (TP < 10 µg/L, TDP < 5 µg/L) with minimal shoreline development. The presence and low-level expression of the *mcy* gene in Tadenac Bay suggests an innate potential for toxin production despite the oligotrophic nature of this system (Table 3). None of the other toxins for which the samples were screened were detected, i.e., anatoxins, saxitoxins, cylindrospermopsins, nodularins (Table 3). In North Bay, a single sample showed the presence of low copy numbers of the saxitoxin gene (*sxtA*), but not the toxin.

### 2.6. Other Bioactive Metabolites Produced by Cyanobacteria

When associated with cyanobacteria, the term ‘toxins’ is generally applied to the small fraction of bioactive metabolites that affect humans and other large vertebrates; however, many bioactive metabolites produced by cyanobacteria have no known effect on human, pet, or livestock health, but are active towards more proximal elements of the food web including competing phytoplankton, bacteria, pathogens (e.g., viruses, chytrids), and grazers (e.g., [13]). These bioactive metabolites, which include a variety of peptides, have been reported previously from some European and US lakes and are known to inhibit metabolic processes (e.g., protease inhibitors) and function in chemical defence [13,19]. Our study represents a first-time analysis of samples from Canadian waters for both cyanobacterial toxins and these other bioactive metabolites. As seen with microcystins, several anabaenopeptins and one cyanopeptolin (1007) as well as their corresponding genes (*apn, oci*) were detected in both TMB and SB (Table 3); however, clear peaks coincident with the *Planktothrix*-dominated DCL were only evident in TMB (Figure 4). Anabaenopeptins (A, B, and F combined) peaked in 2018 at 6.6 µg L^−1^ in the DCL of the meso-oligotrophic TMB, which was comparable to concentrations found in eutrophic surface waters in the United States and Europe (e.g., [51,52,53]). Anabaenopeptins are thought to play a role in chemical defence via their ability to inhibit proteases used by parasites of cyanobacteria, such as chytrids, to digest their hosts [54]. This anti-parasitic activity could afford cyanobacteria in TMB an additional competitive advantage to establish dominance within the DCL.

Our results indicate that these (and potentially other) bioactive metabolites—typically reported from eutrophic systems (e.g., [51])—are also produced in meso-oligotrophic waters. Furthermore, while most studies have concentrated efforts on surface waters where visible blooms of cyanobacteria are most evident, our data demonstrate the potential importance of these semiochemicals in the often-overlooked communities in DCLs.

## 3. Conclusions

We observed the annual formation of persistent DCLs in TMB and SB during the stratification period, with a predominance of the large cyanobacteria *Planktothrix* cf *isothrix*. These deep-living layers were formed near the hypoxic hypolimnion at the intersection of opposing vertical gradients of PAR and nutrients, notably Fe, Mn and PO_4_. Similar DCLs were not observed in the other (13) embayments surveyed along the same coastline, where analogous gradients of PAR and water chemistry were not detected. We also observed the Fe- and PO_4_-precipitating bacteria *Leptothrix* within the DCL of TMB but not SB, suggesting that microbial processes may contribute to differences in their metalimnetic chemical gradients and DCL composition and biomass. In both embayments, the *Planktothrix*-dominated DCLs coincided with measurable levels of cyanobacterial toxins and other bioactive metabolites (most notably microcystins and anabaenopeptins), along with the associated genes. This is the first report of these compounds in DCLs from meso-oligotrophic Canadian lakes, and it merits more focused work to understand if and how these compounds function in the establishment and maintenance of these deep-living communities. The presence of significant numbers of live *Planktothrix* in surficial sediments and overlying water suggest these act as important seed populations to the DCLs, meriting further investigation. Overall, the prevalence of these significant DCLs dominated by large, potentially harmful cyanobacteria across different waterbodies is unknown, and their formation in apparently oligotrophic systems requires more research to understand the driving factors and track their change with changing environmental conditions and anthropogenic development in respective regions.

## 4. Materials and Methods

### 4.1. Study Sites

Fifteen embayments along the eastern shore of Georgian Bay, Lake Huron (Ontario, Canada) were surveyed, with a focus on sheltered embayments of intermediate size and depth (<30 m), particularly those that are reported to regularly undergo stratification and establish pronounced chemical gradients facilitated by hypolimnetic hypoxia (Figure 1) [16,55]. All 15 embayments were sampled during summer (July/August) and autumn (September/October) stratification of 2014 and 2015 (Figure 1 and Figure A1, Table A1). Twelve Mile Bay (TMB) and South Bay (SB) were selected, based on the presence of DCLs, for additional and more detailed sampling and analysis in 2015 in winter (February/March), spring (June), summer (August), and autumn (September/October). Based on the earlier results, TMB and SB were sampled again during stratification in the summer of 2018 (July/August) and analyzed for a broader suite of bioactive metabolites beyond the cyanobacterial toxins commonly measured (Table 3).

### 4.2. Physicochemical Profiles of Water Column

At each sampling, photosynthetically active radiation (PAR; 400–700 nm) was measured with a LI-193 spherical underwater quantum sensor (LI-COR Biosciences, NE, USA) at 0.5 m depth intervals. Measurements were corrected for variance in incident irradiance using a LI-190R quantum sensor (LI-COR Biosciences, NE, USA), and euphotic zone depth calculated (i.e., <1% sub-surface irradiance). A YSI multi-parameter sonde (Xylem Inc., New York, NY, USA) was used to obtain depth profiles of temperature, dissolved oxygen, pH, specific conductivity, redox, and turbidity at each site, while a FluoroProbe (FP) (bbe Moldaenke GmbH, Schwentinental, Germany) was used to measure depth profiles of in situ, fluorescence-based, phytoplankton pigment class-specific chlorophyll (‘green algae’, ‘cyanobacteria’, ’brown’ algae’ (diatoms, chrysophytes and dinoflagellates), and ‘cryptophytes’).

### 4.3. Water Sampling

At each site and date, whole water samples were collected from near-surface (1 m) and bottom (1 m from sediments) using a 10 L Niskin water sampler, subsampled into acid washed polyethylene bottles and kept in the dark at 4 °C until processed (within 24 h). During stratification, samples were also collected from the middle of the thermocline (metalimnion), the epilimnion (1 m), and hypolimnion (1 m from sediments). Samples were also taken at additional depths where DCLs were detected in the FP profiles.

### 4.4. Sediment Sampling

Surficial sediments were sampled using a modified gravity corer (Uwitech, Austria). Overlying water was siphoned and surficial 1.0 cm of the core was extruded on-site, placed in pre-labeled Whirl-Pak^®^ bags, and immediately transported to the laboratory on ice in a dark cooler for microscopic imaging before being frozen in the dark at −20 °C.

### 4.5. Water Quality Analysis

Dissolved and particulate components were analyzed using filtrate from a cellulose acetate filter (0.45 μm pore size, 47 mm diameter) and material collected on a Whatman GF/C filter (1.2 μm nominal pore size, 47 mm diameter), respectively. These included major nutrients (dissolved inorganic and organic carbon (DIC, DOC), particulate organic carbon (POC), nitrate/nitrite (NO_2/3_), ammonium (NH_4_), total dissolved Kjeldahl nitrogen (TKN), particulate organic nitrogen (PON), phosphorus (P), dissolved phosphorus (DP), phosphate (PO_4_)), dissolved silica, and extracted chlorophyll-*a,* all of which were analyzed at the National Laboratory for Environmental Testing (NLET, Burlington, Ontario) using standard methods [56]. Dissolved organic nitrogen (DON) was calculated by subtracting NH_4_^1+^ from TKN.

### 4.6. Extraction and Analysis of Cyanobacterial Bioactive Metabolites

Whole water samples were concentrated onto Whatman GF/C filters (1.2 µm nominal pore size, 47 mm diameter) and stored at −80 °C in the dark until extraction. Cyanobacterial toxins and other bioactive metabolites were extracted in 10 mL of analytical grade aqueous methanol (1:1 *v*/*v*) amended with analytical grade formic acid (0.1%) using probe sonication (three 30 sec pulses) (Fisher Scientific Co. Qsonica Sonicator Q500, Ontario, Canada). Samples were then centrifuged at 3000 rpm for 15 min to pellet debris. A PTFE syringe filter (1.0 µm pore size, 30 mm diameter) attached to a 10 mL gas-tight glass syringe was used to filter the resulting supernatant into a glass vial, which was then evaporated to dryness using nitrogen gas-flow and heat (30 °C). The resulting residue was reconstituted with 1 mL of analytical grade aqueous methanol (1:1 *v*/*v*) and vortexed. A final filtration was done using a PTFE syringe filter (0.45 µm pore size, 15 mm diameter) attached to a gas-tight glass syringe into a 1.5 mL HPLC amber glass vial and stored at −80 °C in the dark until analysis.

Nineteen cyanobacterial peptides and five alkaloids were analyzed by high performance liquid chromatography tandem mass spectrometry (HPLC-MS/MS), as described previously [51]. Certified reference standards of microcystin-LR, -[Dha^7^]LR and nodularin (NOD-R) were purchased from the National Research Council (NRC) of Canada Biotoxins program (Nova Scotia, Canada). Microcystin-RR, -LA, -LF, -YR, -WR, -LY, -LW, -HtyR, and -HilR were purchased from Enzo Life Sciences (New York, NY, USA). Anabaenopeptin-A, -B, and -F; cyanopeptolin-1007, -1021, and -1040; as well as microginin-690 were purchased from MARBIONC (NC, USA). Anatoxin-a fumarate was purchased from Tocris Bioscience (MN, USA) as a racemic mixture. Homo-anatoxin-a was purchased from Abraxis. Cylindrospermopsin was purchased from Enzo Life Sciences. Saxitoxin and neosaxtoxin were purchased from the NRC. Each analyte exceeded 95% purity as per the manufacturer’s certification.

HPLC-MS/MS analysis was performed on a Sciex 4000 QTRAP (AB Sciex, USA) tandem mass spectrometer equipped with a Shimadzu Prominence HPLC. Peptides were chromatographically separated in 20 µL injections of extracts on a Luna C8 column (Phenomenex, CA, USA) using gradient elution where the mobile phase consisted of A (0.1% formic acid and 5 mM ammonium acetate in HPLC grade water) and B (0.1% formic acid and 5 mM ammonium acetate in 95% acetonitrile). The gradient began at 30% B for 3 min, increasing over a linear gradient to 95% B at 9 min, and held at 95% B until 15 min at which point B was returned to the starting condition for 5 min. The mass spectrometer was operated in positive mode using a scheduled multiple reaction monitoring method. Alkaloids were separated by hydrophilic interaction liquid chromatography (HILIC) (SeQuant^®^, 5 μm, 150 × 2.1 mm I.D., EMD Millipore Corporation, MA, USA) with mobile phases of (A) HPLC water with 60 mM formic acid and (B) 100% acetonitrile. Isocratic elution (60% B) was used for the HILIC method. The concentration of target analytes was determined based on a linear regression model of peak area relative to the known concentration of an 8-point calibration curve prepared in 1:1 methanol:water. Regression coefficients of R > 0.98 were accepted at an accuracy of >90% at each calibration level.

### 4.7. Taxonomic Identification and Enumeration

Unfiltered sample aliquots of 100 mL were preserved in Lugol’s iodine solution (2% *v*/*v*) for later taxonomic identification, abundance, and biomass using the Utermöhl technique [57].

### 4.8. DNA Extraction and PCR Amplification

Water samples were filtered onto 0.22 µm pore size, 47 mm polycarbonate filters and stored at −80 °C for DNA extraction. Samples were extracted using DNeasy PowerWater kit (Qiagen, Canada) following the manufacturer’s protocol.

The availability of a commercial multiplex, quantitative PCR (qPCR) kit for genes of cylindrospermopsin (*cyrA*), microcystin/nodularin (*mcyE*/*ndA*), and saxitoxin (*sxtA*) facilitated the simultaneous measurement of respective gene copy numbers. The qPCR assays were performed using the Phytoxigene^TM^ kit (Diagnostic Technology, Australia) according to the manufacturer’s protocol. Briefly, the lyophilized master mix was first spun down and then reconstituted in PCR-grade water. Each qPCR reaction consisted of 20 µL of the reconstituted master mix and 5 µL of template DNA (10–50 ng) and was carried out in a Bio-Rad CFX96 cycler (Bio-Rad, USA). The cycling conditions consisted of an initial denaturation at 95 °C for 2 min, followed by 40 cycles of denaturation at 95 °C for 15 sec and annealing-extension at 60 °C for 30 s. Gene copies per sample were calculated using a standard curve (target gene copy number vs. Ct) determined for each target gene. Standard curves for all target genes were constructed using standards purchased from the same manufacturer (correlation coefficient and efficiency: *mcyE*/*ndA*: R^2^ = 0.999; E = 100.7%; *cyrA*: R^2^ = 0.999; E = 100.7%; *sxtA*: R^2^ = 1.000; E = 102.1%).

All samples were screened by conventional endpoint PCR for the presence of anatoxin-a synthesis gene and several synthesis genes of oligopeptides know to be bioactive metabolites: aeruginoside, anabaenopeptin, cyanopeptolin, microcystin, microginin, and prenylagaramide. PCR was performed in a total volume of 25 µL composed of 5 µL of GoTaq 10X buffer (Promega), 0.5 µL of dNTPs (10 mM each), 0.5 µL each forward and reverse primers (10 µM), 0.125 µL GoTaq Polymerase (5 units µL^−1^), 2 µL of template DNA (10–50 ng), and 16 µL of nuclease-free water (see Table A2 for primer sequences). The thermal cycling condition consisted of an initial denaturation step at 95° C for 3 min followed by 35 cycles of denaturation at 95° C for 1 min, annealing for 30 sec (variable annealing temperature; see Table A2), and extension at 72° for 30 s. PCR products were evaluated on a 2% agarose gel.

Anabaenopeptin gene copies were quantified using digital PCR (dPCR). Each dPCR mixture consisted of 7.5 µL of QuantStudio 3D digital PCR master mix v.2 (Thermo Fisher Scientific, Canada), 1.4 µL of each 900 nM forward and reverse primer, 0.75 µL of 250 nM probe, 2 µL of DNA extract, and 195 µL nuclease-free water. The reaction mixtures were loaded into a QuantStudio 3D digital PCR 20K chip v2 (Thermo Fisher Scientific, Canada) using a QuantStudio 3D chip loader (Thermo Fisher Scientific, Canada). The reactions were carried out in a ProFlex thermocycler (Thermo Fisher Scientific, Canada) using the following cycling conditions: 96 °C for 10 min, 40 cycles of 60 °C for 2 min and 98 °C for 30 s, and a final step of 60 °C for 2 min. The chips were read on a QuantStudio 3D digital PCR instrument and the results analyzed using the QuantStudio AnalysisSuite software (V 3.2, 2019) (Thermo Fisher Scientific, Canada).

## Figures and Tables

**Table 1:** Depth of mixed (Z_mix_) and euphotic zones (Z_eu_) during mixed and stratified conditions and presence (Y: Yes/N: No) of deep-chlorophyll layer (DCL) in different embayments of Georgian Bay in the spring (June) and summer (August) of 2015. During mixed conditions in spring (June), Z_mix_ was equivalent to the site depth (Z_site_).

		Mixed	Stratified			
Site	Z_site_ (m)	Z_eu_ (m)	Z_eu_ (m)	Z_mix_ (m)	Z_eu_:Z_mix_	DCL (m)
Byng Inlet	5	3.0	-	5	-	N
Sturgeon Bay North	14	2.4	4.5	8	0.6	N
Sturgeon Bay South	6	-	-	-	-	N
Deep Bay	15	2.7	3.7	8	0.5	N ^a^
Woods Bay	8	-	-	-	-	N
Twelve Mile Bay	12	3.4	9.1	7	1.3	Y (~9)
Tadenac Bay	24	6.5	5.3	11	0.5	N
Go Home Bay	10	3.6	-	-	-	N
Longuissa Bay	9	3.8	4.7	8	0.6	N
Cognashene Lake	16	3.9	9.5	7	1.4	N
Musqwash Bay West	30	-	-	-	-	N
Musqwash Bay East	20	-	-	-	-	N
North Bay	18	3.2	6.4	8	0.8	N
South Bay	10	3.9	5.7	6	1.0	Y (~7)
Honey Harbour	7	4.6	3.6	7	0.5	N

^a^ Not detected during this study but reported in 2012 and 2014 by [16].

**Table 2:** Nutrient concentrations (µg/L) and depth ratios in the hypolimnion (Hypo) and epilimnion (Epi) in each embayment (DON = dissolved organic nitrogen). Data are based on the summer 2015 sampling expedition during stratification.

Nutrient	Twelve Mile Bay			South Bay		
	Hypo	Epi	Hypo: Epi	Hypo	Epi	Hypo:Epi
P	15	14	1	232	13	18
Fe	127	8	16	4160	10	408
NO_2/3_	11	9	1	6	8	0.8
Si	2.5	1.3	2	8	4	2
Mn	210	0.3	660	1020	0.5	2040
Co	0.2	0.01	20	0.3	0.01	26
NH_4_	20	5	4	589	5.5	107
SO_4_	10	11	1	2.3	7.7	0.3
DON	212	196	1	367	299	1
PO_4_	0.4	0.8	0.5	172	0.7	246

**Table 3:** Major bioactive metabolites (right column) ^1^ and corresponding genes (left column) ^2^ (detect “+”; non-detect “-”; not measured “NM”; or maximum concentration (µg/L), any depth) in water samples from embayments along eastern Georgian Bay during 2014, 2015, and 2018. MC: Microcystins, NOD: Nodularin, CYN: Cylindrospermopsins, STX: Saxitoxins, ATX: Anatoxins, APT: Anabaenopeptins, CPT: Cyanopeptolins, MG: Microginins. NOD, CYN, and ATX were not detected in any of the samples.

Sites	MCs		STXs		APTs		CPTs		MGs	
Byng Inlet	-	-	-	-	-	-	-	-	-	-
Sturgeon Bay North	+	0.01	-	-	-	-	-	-	-	-
Sturgeon Bay South	NM	NM	NM	NM	NM	NM	NM	NM	NM	NM
Deep Bay	-	-	-	-	-	-	-	-	-	-
Woods Bay	NM	NM	NM	NM	NM	NM	NM	NM	NM	NM
Twelve Mile Bay ^3^	+	0.4	-	-	+	6.6	+	0.001	-	-
Tadenac Bay	+	0.01	-	-	-	-	-	-	-	-
Go Home Bay	-	-	-	-	-	-	-	-	-	-
Longuissa Bay	-	-	-	-	-	-	-	-	-	-
Cognashene Lake	-	-	-	-	-	-	-	-	-	-
Musqwash Bay West	NM	NM	NM	NM	NM	NM	NM	NM	NM	NM
Musqwash Bay East	NM	NM	NM	NM	NM	NM	NM	NM	NM	NM
North Bay	-	-	+	-	-	-	-	-	-	-
South Bay	+	0.03	-	-	+	0.01	+	0.001	+	-
Honey Harbour	+	0.04	-	-	-	-	-	-	-	-

^1^ ELISA and PPIA results verified by LC-MS/MS. ^2^ PCR primers listed in Table A2 (adapted from [18,50]. ^3^ Peaks in Twelve Mile Bay were measured in 2018 and the highest concentrations of these bioactive metabolites (and their genes) co-occurred with increasing biomass, notably of *Planktothrix* cf. *isothrix*, at the depth of the deep chlorophyll layer (Figure 4, Table S1, 2018 data).

## Data Availability

Data is contained within the article or supplementary material.

## Supplementary Materials

The following are available at https://www.mdpi.com/article/10.3390/toxins13070445/s1, Table S1: Taxonomic identification and enumeration of phytoplankton in Twelve Mile Bay and South Bay embayments of Georgian Bay, Lake Huron (Ontario, Canada).

## Appendix A

**Table A1:** Coordinates and depths of sites under study in Georgian Bay, Lake Huron (Ontario, Canada). All 15 embayments were sampled during summer (July/August) and autumn (September/October) stratification of 2014 and 2015. Twelve Mile Bay and South Bay were selected (based on presence of DCLs) for additional and more detailed sampling and analysis in 2015 in winter (February/March), spring (June), summer (August), and autumn (September/October). TMB and SB were again sampled during stratification in the summer of 2018 (July/August) and analyzed for a broader suite of bioactive metabolites beyond the cyanobacterial toxins commonly measured (Table 3).

Site	Latitude	Longitude	Depth (m)
Byng Inlet	45.77056	−80.56836	3
Sturgeon Bay North	45.6133	−80.4325	14
Sturgeon Bay South	45.6055	−80.4096	6
Deep Bay	45.3953	−80.2236	15
Woods Bay	45.13785	−79.9894	8
Twelve Mile Bay	45.0838	−79.946	12
Tadenac Bay	45.0588	−79.9775	24
Go Home Bay	44.9913	−79.9376	10
Longuissa Bay	44.9613	−79.8884	8
Cognashene Lake	44.9510	−79.9186	16
Musqwash Bay West	44.9520	−79.8787	30
Musqwash Bay East	44.9483	−79.85006	20
North Bay	44.8919	−79.7925	18
South Bay	44.8762	−79.7857	10
Honey Harbour	44.8770	−79.8263	7

**Table A2:** Primers used to amplify regions of genes coding for cyanobacterial toxins and other bioactive metabolites (oligopeptides).

Gene Loci	Primer ^1^	Sequence (5′-3′) ^2^	Amplicon Length (bp)	Annealing Temperature (°C)
Aeruginoside				
aerD	aerD-F	GAAACCAGTAGTGAACAGACCTTAAATTATC	493	60
	aerD-R	GACCAACTCATCTCAATTTTCCC		
Anabaenopeptin				
apnD	apnDTe-F	GACACGCCTTCTATTTTCGAGA	581	60
	apnDTe-R	CGCGAAATAAAACAATAGGGG		
apnC	apnNMT-F	CGTGCAGATGATGACCTATCCA	470	55
	apnNMT-R	AAGGTTCGCAATACTTCAGGGTT		
Anatoxin ^3^				
anaC	anaC-gen-F2	TCTGGTATTCAGTMCCCTCYAT	366	58
	anaC-gen-R2	CCCAATARCCTGTCATCAA		
Cyanopeptolin				
ociC	cptDTe-F	GATCTCTATCAACAGTTTGGAGCAA	690	61
	cptDTe-R	ACTGTTCGGCTAACACTTGAACAT		
ociB	ociB-F	TGGTTTTTAGATCAATTTGAGTCCG	512	66
	ociB-R	CCACTGTTTTTGCCAAAGAGTG		
Microcystin				
mcyC	mcyCTe-F	TTACAAGCGATGAATCTCATGG	503	60
	mcyCTe-R	GGGATTTAATAAGAAACCATCAACC		
mcyE	Hep-F	TTTGGGGTTAACTTTTTTGGGCATAGTC	466	60
	Hep-R	AATTCTTGAGGCTGTAAATCGGGTTT		
Microginin				
micD	mgnTe-F	TGGTCAATGGGAGGAGTGATAG	514	60
	mgnTe-R	CTGTAGTGATCTCCACTAATCCATTG		
micA	mcnA-F	AAACCCTTTGATTTGAGCCAG	428	60
	mcnA-R	CAGCAAGGTACAGCCCTGTT		
Prenylagaramide				
pagA	acyA-F	CCCTGGAAAAGATATTTTAGGAGC	489	60
	acyA-R	ATTTGAAGTACGGTTTGACATGG		

^1^ Primers for cylindrospermopsin, nodularin, and saxitoxin are not listed here since these toxins were only measured using the Phytoxigene^TM^ kit (Diagnostic Technology, Australia) and the primers are included in the kit’s master mix. ^2^ [18]. ^3^ [50].
